# Citropin 1.1 Trifluoroacetate to Chloride Counter-Ion Exchange in HCl-Saturated Organic Solutions: An Alternative Approach

**DOI:** 10.1007/s10989-017-9611-7

**Published:** 2017-07-12

**Authors:** Karol Sikora, Damian Neubauer, Maciej Jaśkiewicz, Wojciech Kamysz

**Affiliations:** Department of Inorganic Chemistry, Faculty of Pharmacy, Medical University of Gdansk, Al. Gen. J. Hallera 107, 80-416 Gdansk, Poland

**Keywords:** Citropin 1.1, Counter-ion exchange, Organic solvents, Trifluoroacetate, Chloride

## Abstract

In view of the increasing interest in peptides in various market sectors, a stronger emphasis on topics related to their production has been seen. Fmoc-based solid phase peptide synthesis, although being fast and efficient, provides final products with significant amounts of trifluoroacetate ions in the form of either a counter-ion or an unbound impurity. Because of the proven toxicity towards cells and peptide activity inhibition, ion exchange to more biocompatible one is purposeful. Additionally, as most of the currently used counter-ion exchange techniques are time-consuming and burdened by peptide yield reduction risk, development of a new approach is still a sensible solution. In this study, we examined the potential of peptide counter-ion exchange using non-aqueous organic solvents saturated with HCl. Counter-ion exchange of a model peptide, citropin 1.1 (GLFDVIKKVASVIGGL-NH_2_), for each solvent was conducted through incubation with subsequent evaporation under reduced pressure, dissolution in water and lyophilization. Each exchange was performed four times and compared to a reference method—lyophilization of the peptide from an 0.1 M HCl solution. The results showed superior counter-ion exchange efficiency for most of the organic solutions in relation to the reference method. Moreover, HCl-saturated acetonitrile and *tert*-butanol provided a satisfying exchange level after just one repetition. Thus, those two organic solvents can be potentially introduced into routine peptide counter-ion exchange.

## Introduction

The global market of synthetic peptides is based mainly on such sectors as pharmaceutical, cosmetic or food industry and in these fields peptides are thought to be primary targets of new, modified compounds. Generally, peptides are considered to be poor candidates for drugs due to their low bioavailability and their rapid metabolism (Marx 2005). However, since new synthetic strategies as well as alternative routes of administration have been developed, a large number of peptide-based drugs are now being merchandised (Vlieghe et al. 2010). Cosmetic and cosmeceutic industry also relies on the use of synthetic peptides and lipopeptides as dermatologically active molecules or carrier agents for skin applications (Goebel and Neubert 2008; Silva et al. 2008). For example a palmitoyl derivative of polypeptide interferon α exhibits greater skin penetration than a single polypeptide, while palmitoyl signal peptide from a pro-collagen I fragment (palmitoyl pentapeptide-4, Pal-KTTKS) enhances the skin regeneration (Foldvari et al. 1998; Robinson et al. 2005; Lupo and Cole 2007; Gorouhi and Maibach 2009).

In industry as well as in academic laboratories peptide, synthesis is performed on a solid support. Solid-phase peptide synthesis (SPPS), particularly with Fmoc chemistry, is directly related to the use of trifluoroacetic acid (TFA). It is used as a cleavage reagent—to release peptide from the resin. Additionally, crude peptides are usually purified with reverse-phase high-performance liquid chromatography (RP-HPLC) and TFA is used as a component of the mobile phase. Considering all these facts it is not surprising that the final product contains significant amounts of trifluoroacetate ions (TFA^−^). In general, anions interact with cationic peptide moieties such as the amino group (lysine and *N*-terminus), and the guanidine group (arginine), and imidazole (histidine) owing to electrostatic forces.

The presence of TFA^−^ both adsorbed in lyophilizate and directly bound to peptide molecule can affect biological and physicochemical properties. There are several reports on the high toxicity of TFA^−^ towards cells, i.e. by inhibition of proliferation (Cornish et al. 1999). Moreover, TFA in excess can interact with molecules by pH change and modification of peptides conformation (Shen et al. 1994; Wada et al. 2003; Nick Pace et al. 2004). It should also be taken into account that TFA^−^ may influence structural analysis, i.e. by interfering with amide band in IR absorption spectra or by alternating conformation in CD experiments (Andrushchenko et al. 2007). Due to those effects, the type of counter-ion should be considered both in the in vitro and in vivo studies, and commercial use (Pini et al. 2012).

To avoid those problems, TFA^−^ should be exchanged for a biocompatible ion, for example, chloride or acetate. Various procedures of counter-ion exchange are described in the literature, for instance dissolution of the peptide in acid solution and usually 0.1 M HCl_aq_ lyophilization (Andrushchenko et al. 2007); RP-HPLC with acetic acid or HCl as an ion-pairing agent; ion-exchange resin with proper counter-ion; washing peptides in dialysis membranes and deprotonation/reprotonation of basic amino acid residues (Roux et al. 2008). However, most of techniques are time-consuming and substantially reduce the peptide yields. Moreover, to characterize synthetized peptides, there is a need for analytical procedures to determine the level of TFA^−^ and other ions in samples. Different techniques are applied such as ion chromatography (IC), capillary electrophoresis, IR and NMR spectroscopy (Kaiser and Rohrer 2004; Roux et al. 2008).

Dissociation and acid–base reactions are strongly influenced by external factors, including the type of solution and temperature. Moreover, diverse solvents may act differently on basic moieties and acid molecules by changing the protonation state and therefore influencing the ion exchange process (Porras et al. 2001; Psurek and Scriba 2003). Factors that should be considered are: solvent dielectric constant and the ability to form hydrogen bonds (Sarmini and Kenndler 1999). There are significant changes in dissociation equilibrium and pK_a_ values, for instance, acetic acid in water has a pK_a_ of 4.73 while in methanol and acetonitrile the values are 9.7 and 22.3 respectively (Sarmini and Kenndler 1999). Similar change in the pK_a_ is observed for other acids, i.e. for TFA which in water has a pK_a_ of 0.2, while 12.65 in acetonitrile (Eckert et al. 2009). Those fact suggest that anion exchange reaction may be improved in a non-aqueous environment.

In the following study new approach for TFA^−^ exchange to chlorides is presented. Organic solvents saturated with HCl were applied. The model peptide, citropin 1.1 was synthetised by SPPS using Fmoc strategy. The TFA^−^ exchange to chlorides was followed by IC.

## Materials and Methods

### Peptide Synthesis

Citropin 1.1 (GLFDVIKKVASVIGGL-NH_2_), CAMEL (KWKLFKKIGAVLKVL-NH_2_), LL-37 (LLGDFFRKSKEKIGKEFKRIVQRIKDFLRNLVPRTES), pexiganan (GIGKFLKKAKKFGKAFVKILKK-NH_2_), and temporin A (FLPLIGRVLSGILNH_2_) were synthesized on the solid support (Rink Amide or Wang resin) with Fmoc/tBu methodology. All reactions were run using a CEM microwave synthesizer (Liberty Blue) to provide higher efficiency compared to conventional methodology (Rizzolo et al. 2011). Coupling reactions were carried out by activation with DIC (*N,N′*-diisopropylcarbodiimide) in DMF (*N,N*-dimethylformamide). OxymaPure was applied to suppress racemization instead of HOBt due to superior coupling efficiencies (Subirós-Funosas et al. 2009). Single deprotection step was accomplished in a 20% piperidine solution in DMF. Deprotection was performed at 75 °C using 30 W for 3 min, whereas the coupling steps were performed at 75 °C, using 30 W for 5 min. The reagents were used in a fourfold excess according to the substitution level of the resin. A mixture of TFA, TIS (triisopropylsilane) and water (96:2:2, v/v) was used to cleave a peptide from the resin. This reaction was performed for 90 min under stirring. The crude peptides were lyophilized and subsequently purified by RP–HPLC. Acetonitrile and water both containing 0.1% of TFA were used as a mobile phase.

### LC–MS Analysis

The purity and identity of the peptides was confirmed by LC–MS analysis. RP–HPLC system was used—Waters Alliance e2695 system with Waters 2998 PDA and Acquity QDA detectors (software—Empower^®^3). All analyses were carried out on a Waters XBridge™ Shield RP-18 column (4.6 × 150 mm, 3.5 µm particle size, 130 Å pore size). Samples (10 µL) were analyzed with a linear 10–90% acetonitrile gradient in deionized water over 15 min at 25.0 ± 0.1 °C. The mobile phase flow rate was 0.5 mL/min. Both eluents contained 0.1% (v/v) of formic acid. Mass analysis and UV detection at 214 nm were used.

### Counter-Ion Exchange

Exchange of the TFA^−^ ion in 0.1 M HCl solution of was used as a reference method. This approach is based on a few steps, as follows—dissolution of the peptide in a 0.365% (w/w) HCl solution, incubation for 5 min, and further lyophilization. Exchange procedure was repeated four times, and after each step a sample was collected for IC and LC–MS analysis. Finally, peptide samples were lyophilized from water to remove excess of chlorides.

The method with organic solvents was different, i.e. evaporation under reduced pressure using a rotary evaporator at 40 °C after the incubation step. Before use, all organic solvents were dried using molecular sieves. Subsequently, the solvents were saturated with gaseous HCl produced in appropriate apparatus through reaction of sulphuric acid with sodium chloride. Saturation level was determined by weight. Organic solvents used in this study were acetonitrile, dichloromethane, ethyl acetate, 2-propanol, methanol, *n*-butanol, and *tert*-butanol.

**Fig. 1:**
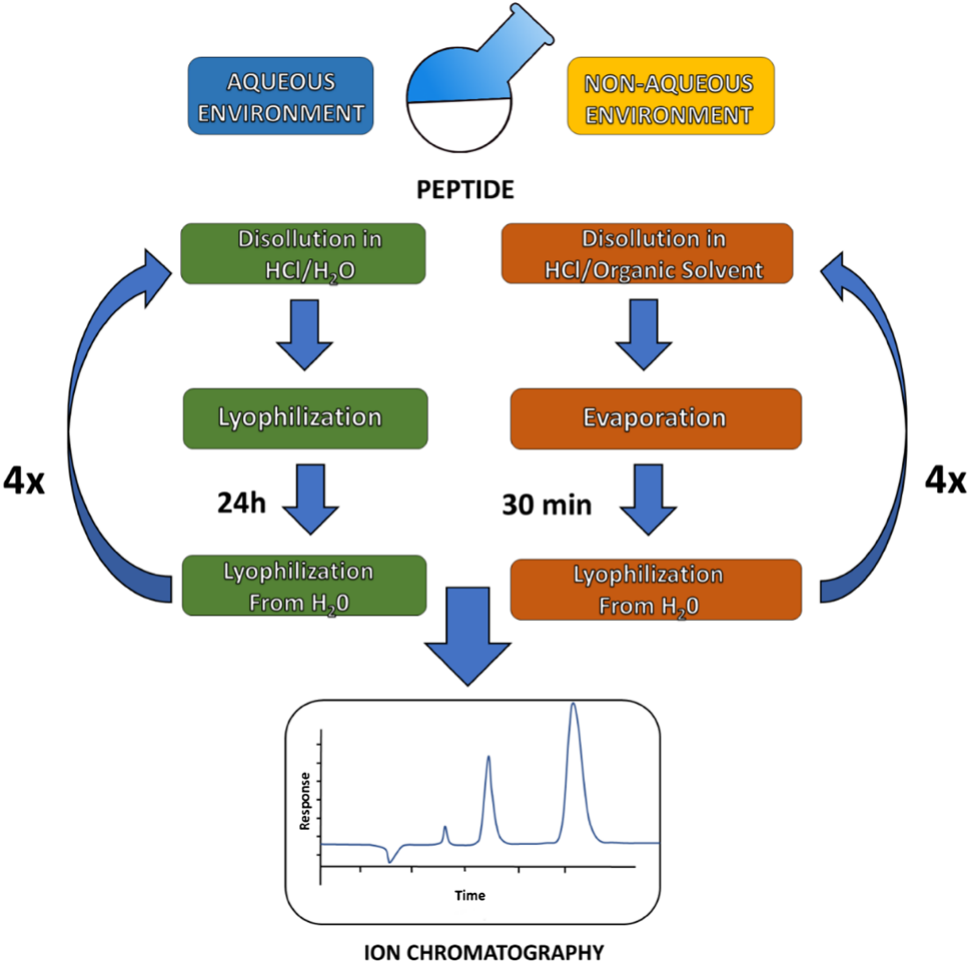
Schematic diagram of counter-ion exchange

During exchange peptide concentration in each solution was 1 mg/mL. Counter-ion exchange was performed four times for all solvents. After each exchange sample of peptide was collected and analyzed. Both procedures are presented in Fig. 1.

### Ion-Chromatography

Each time the counter-ion content was determined using ion chromatography (Dionex ICS5000+). Analyses were performed with isocratic elution (4.5 mM Na_2_CO_3_ and 1.4 mM NaHCO_3_ in water), a flow rate of 1.2 mL/min, and injection volume of 20 µL. All of the tested samples were dissolved in water to obtain concentration of 0.5 mg/mL. Ions were detected by suppressed conductivity with ASRS 300—anion self-regenerating suppressor and suppressor current −31 mA. Column characteristics—Dionex IonPac AS22, dimensions 4.0 × 250 mm. Column compartment temperature was set at 30 °C and conductivity detector temperature at 35 °C. The validation of the method was performed in accordance with the ICH guidelines Q2(R1) (ICH 2005).

## Results and Discussion

In the following study, citropin 1.1 was used as a model peptide. The compound was selected as it contains a variety of side chains characteristics: hydrophobic, hydrophilic, basic (primary amine), acidic, and aromatic. Moreover, it possesses a positive net charge (+2). After synthesis and purification, identity and purity of the compound was determined by LC–MS analysis. Subsequently, the counter-ion content (TFA^−^) was determined using ion chromatography (IC). The examined peptide contained 189.94 µg of TFA^−^ per 1 mg of lyophilizate. TFA^−^ counter-ions were exchanged for Cl^−^ using two different methods: in aqueous environment and in organic solvents. The method was tested on citropin 1.1; however, selected solutions were also successfully applied for other peptides (unpublished data).

As a reference method, the exchange by multiple lyophilizations of peptide dissolved in 0.1 M HCl (**A**, Table 1) were performed. After each step, samples were analyzed using LC–MS and IC. By this method, a gradual increase in Cl^−^ and simultaneous decrease in TFA^−^ in peptide samples were observed. First exchange resulted in the 87 mol% of chlorides and after four repetitions a 96 mol% was achieved (Table 1). According to LC–MS no peptide degradation was observed. Interestingly, similar results were obtained for other peptides (pexiganan and temporin A) in previous works (Mrozik et al. 2012).

**Table 1:** Counter-ion content after each exchange

Solution		Counter-ion [mol%]							
		Repetition							
		1		2		3		4	
		Cl^−^	TFA^−^	Cl^−^	TFA^−^	Cl^−^	TFA^−^	Cl^−^	TFA^−^
A	0.1 M HCl (0.365%)	**87**	**13**	93	7	94	6	96	4
B	0.5% HCl in acetonitrile	**98**	**2**	98	2	98	2	98	2
C	2.0% HCl in dichloromethane	**90**	**10**	92	8	94	6	96	4
D	1.4% HCl in ethyl acetate	**77**	**23**	85	15	86	14	98	2
E	1.5% HCl in 2-propanol	**97**	**3**	98	2	99	1	99	1
F	2.5% HCl in methanol	**98**	**2**	99	1	99	1	99	1
G	1.5% HCl in *n*-butanol	**97**	**3**	98	2	99	1	99	1
H	1.5% HCl in *tert*-butanol	**98**	**2**	99	1	99	1	99	1

Bold values indicate efficiency of proposed method after just one repetition

In contrast to the reference method, the exchange performed using organic solvents provided almost complete substitution of TFA^−^ to Cl^−^ after first repetition. Results of IC analysis are summarized in Table 1. Satisfactory results of exchange were obtained for solutions, as follows: (**B**) acetonitrile, (**E**) 2-propanol, (**F**) methanol, (**G**) *n*-butanol and (**H**) *tert*-butanol. Among those solutions a single repetition of the procedure provides 97–98 mol% of chlorides. Considering no difference between samples obtained after treatment with solution **B**, a single repetition seems to be an optimal choice (98 mol%). In the case of solutions **H** and **F**, two repetitions of the procedure provided successful exchange (99 mol%). The same result was achieved after a third repetition with solutions **G** and **E**. The lowest exchange rate was observed for the ethyl acetate (**D**) and dichloromethane (**C**) solutions. Repetition of the procedure allowed to reach 98 mol% of Cl^−^ and 96 mol%, respectively. The procedure with methanol (**F**) was associated with serious side reaction such as peptide esterification and the LC–MS analyses confirmed esterification of citropin 1.1 in our study. It is well known that esterification may occur in the presence of a strong acid, alcohol and a peptide carboxyl moiety. Presumably, the side-chain of aspartic acid was esterified. The measured mass was 1629.02 Da and the calculated, respectively monoisotopic and average—1628.00, 1629.00 Da. Furthermore, a side-product with a mass of 1643.66 Da was identified in methanolic sample what indicated the C-terminal amide methanolysis (calculated mass—monoisotopic 1643.00 Da, and average 1644.01 Da). No esterification was observed when the counter-ion exchange was performed in the remaining alcohols (2-propanol, *n*-butanol, *tert*-butanol). Important issue of counter-ion exchange is also peptide solubility. Although citropin 1.1 exhibited good solubility in applied solvents; this aspect should always be considered. A wide range of organic solvents with different properties (dielectric constant, proton acceptor or donor character, and dipole moment) may provide possibility to dissolve a variety of peptides.

During prolonged storage in acidic media some serious side reactions can occur, including peptide bond hydrolysis, oxidation or isomerization (Oliyai and Borchardt 1993; Reubsaet et al. 1998). Factors such as: time, temperature, and pH of solution should be taken into account. It is well known that considerable amount of D-isomers, as high as 10%, are formed during hydrolysis when 6 M HCl is used for amino acid analysis (Kaiser and Benner 2005). In our study no side reactions were observed, except esterification in methanol solution. Relatively low temperature, short incubation time, and fast removal of acidic solution in proposed procedure minimized the risk of side reactions. We did not observe any products of peptide bond hydrolysis (LC–MS analysis). Furthermore, no epimerization was noticed, as it can be monitored by HPLC analysis (Huang et al. 2014)(Reubsaet et al. 1998).

We have also tried to apply this method for exchange TFA^−^ for acetate anions. Mixtures of acetic acid with water and organic solvents were used. All procedures were carried out analogously to that described above. We did not observe any peptide degradation. In contrast to chlorides, exchange level to acetate anions was far from satisfactory for all solvents. Presumably, the reason is the fact that acetic acid is weaker than trifluoroacetic acid with pKa values 4.76 and 0.2 in water in normal conditions, respectively (Sarmini and Kenndler 1999; Eckert et al. 2009). Moreover, organic solvents did not provide any improvement in counter-ion exchange.

The presented method of TFA salts exchange to hydrochlorides is routinely used in our laboratory for a variety of peptides for a period of time. Among others, the successful exchange of counter-ions was carried for other peptides, such as: pexiganan, CAMEL, temporin A, LL-37 and lipopeptides (i.e. Pal-KKK-NH_2_). In our practice, a saturated acetonitrile is used preferably, but alcohols (*tert-*butanol and *n*-butanol) are also applicable. Results of counter-ion exchange using 0.5% HCl in acetonitrile are presented in Table 2. Almost in all cases, two repetitions resulted in successful exchange of TFA^−^ for Cl^−^. Moreover, no side reactions were observed.

**Table 2:** Counter-ion content after exchange using 0.5% HCl in acetonitrile. Procedure was repeated two times

	Counter-ion [mol%]	
Peptide	Cl^−^	TFA^−^
CAMEL	99	1
Pexiganan	99	1
LL-37	99	1
Temporin A	97	3

## Conclusions

In this study, the method of peptide counter-ion exchange was modified and improved. The method is based on the use of organic solvents, saturated with gaseous HCl. Proposed procedure is less time-consuming compared to the classic, reference method (a single repetition, approx. 48 vs. 24 h). In contrast to the aqueous environment, the repetition of the procedure in organic solvent takes only 30 min and is characterized by exchange a higher rate. Although organic solvent solutions have been found to be an excellent alternative, it seems that exchange reactions conducted in acetonitrile (**B**) and *tert*-butanol (**H**) solutions gave slightly superior results. The applied organic solutions allowed to obtain satisfactory levels of exchange, provided good peptide solubility, and additionally, could be readily evaporated. Further studies should consider the use of organic HCl solutions and wide-range of acid concentration in order to optimize the methods and to compare the exchange using particular solvents. This approach may shed light on the particular impact of environment on the peptide counter-ion exchange.

## References

[CR1] Andrushchenko VV, Vogel HJ, Prenner EJ (2007). Optimization of the hydrochloric acid concentration used for trifluoroacetate removal from synthetic peptides. J Pept Sci.

[CR2] Cornish J, Callon KE, Lin CQ (1999). Trifluoroacetate, a contaminant in purified proteins, inhibits proliferation of osteoblasts and chondrocytes. Am J Physiol.

[CR3] Eckert F, Leito I, Kaljurand I (2009). Prediction of acidity in acetonitrile solution with COSMO-RS. J Comput Chem.

[CR4] Foldvari M, Attah-Poku S, Hu J (1998). Palmitoyl derivatives of interferon α: potential for cutaneous delivery. J Pharm Sci.

[CR5] Goebel A, Neubert RHH (2008). Dermal peptide delivery using colloidal carrier systems. Skin Pharmacol Physiol.

[CR6] Gorouhi F, Maibach HI (2009). Role of topical peptides in preventing or treating aged skin. Int J Cosmet Sci.

[CR7] Huang Y, Pan L, Zhao L (2014). Structure-guided RP-HPLC chromatography of diastereomeric α-helical peptide analogs substituted with single amino acid stereoisomers. Biomed Chromatogr.

[CR8] ICH (2005) Validation of analytical procedures: text and methodology Q2 (R1). in: International conference on Harmonization. pp 1–13

[CR9] Kaiser K, Benner R (2005). Hydrolysis-induced racemization of amino acids. Limnol Oceanogr Methods.

[CR10] Kaiser E, Rohrer J (2004). Determination of residual trifluoroacetate in protein purification buffers and peptide preparations by ion chromatography. J Chromatogr A.

[CR11] Lupo MP, Cole AL (2007). Cosmeceutical peptides. Dermatol Ther.

[CR12] Marx V (2005). Watching peptide drugs grow up. Chem Eng News.

[CR13] Mrozik W, Markowska A, Guzik Ł (2012). Determination of counter-ions in synthetic peptides by ion chromatography, capillary isotachophoresis and capillary electrophoresis. J Pept Sci.

[CR14] Nick Pace C, Trevino S, Prabhakaran E, Martin Scholtz J (2004). Protein structure, stability and solubility in water and other solvents. Philos Trans R Soc B Biol Sci.

[CR15] Oliyai C, Borchardt RT (1993). Chemical pathways of peptide degradation. VII. Solid State chemical instability of an aspartyl residue in a model hexapeptide. Pharm Res.

[CR16] Pini A, Lozzi L, Bernini A (2012). Efficacy and toxicity of the antimicrobial peptide M33 produced with different counter-ions. Amino Acids.

[CR17] Porras SP, Riekkola M-LL, Kenndler E (2001). Capillary zone electrophoresis of basic analytes in methanol as non-aqueous solvent mobility and ionisation constant. J Chromatogr A.

[CR18] Psurek A, Scriba GKE (2003). Peptide separations and dissociation constants in nonaqueous capillary electrophoresis: comparison of methanol and aqueous buffers. Electrophoresis.

[CR19] Reubsaet JL, Beijnen JH, Bult A (1998). Analytical techniques used to study the degradation of proteins and peptides: chemical instability. J Pharm Biomed Anal.

[CR20] Rizzolo F, Testa C, Lambardi D (2011). Conventional and microwave-assisted SPPS approach: a comparative synthesis of PTHrP(1-34)NH2. J Pept Sci.

[CR21] Robinson LR, Fitzgerald NC, Doughty DG (2005). Topical palmitoyl pentapeptide provides improvement in photoaged human facial skin. Int J Cosmet Sci.

[CR22] Roux S, Zékri E, Rousseau B (2008). Elimination and exchange of trifluoroacetate counter-ion from cationic peptides: a critical evaluation of different approaches. J Pept Sci.

[CR23] Sarmini K, Kenndler E (1999). Ionization constants of weak acids and bases in organic solvents. J Biochem Biophys Methods.

[CR24] Shen C-L, Fitzgerald MC, Murphy RM (1994). Effect of acid predissolution on fibril size and fibril flexibility of synthetic beta-amyloid peptide. Biophys J.

[CR25] Silva R, Little C, Ferreira H, Cavaco-Paulo A (2008). Incorporation of peptides in phospholipid aggregates using ultrasound. Ultrason Sonochem.

[CR26] Subirós-Funosas R, Prohens R, Barbas R (2009). Oxyma: an efficient additive for peptide synthesis to replace the benzotriazole-based HOBt and HOAt with a lower risk of explosion. Chem A Eur J.

[CR27] Vlieghe P, Lisowski V, Martinez J, Khrestchatisky M (2010). Synthetic therapeutic peptides: science and market. Drug Discov Today.

[CR28] Wada K, Mizuno T, Oku J-I, Tanaka T (2003). pH-induced conformational change in an α-helical coiled-coil is controlled by his residues in the hydrophobic core. Protein Pept Lett.

